# Supplementary material for: HER2-Specific Targeted Toxin DARPin-LoPE: Immunogenicity and Antitumor Effect on Intraperitoneal Ovarian Cancer Xenograft Model
Source: Int J Mol Sci. 2019 May 15;20(10):2399. doi: 10.3390/ijms20102399 (PMC6567818; doi:10.3390/ijms20102399)
Supplement: Supplementary file 1 [file ijms-20-02399-s001.pdf]

SUPPLEMENTARY FIGURES

```
        10         20         30         40         50         60
DLGKKLLEAA RAGQDDEVRI LMANGADVNA HDFYGITPLH LAANFGHLEI VEVLLKHGAD

        70         80         90        100        110        120
VNAFDYDNTP LHLAADAGHL EIVEVLLKYG ADVNASDRDG HTPLHLAARE GHLEIVEVLL

       130        140        150        160        170        180
KNGADVNAQD KFGKTAFDIS IDNGNEDLAE ILQEFKASGG RHRQPRGWEQ LPTGAEFLGD

       190        200        210        220        230        240
GGDVSFSTRG TQNWTVERLL QAHAQLEERG YVFVGYHGTF LEAAQSIVFG GVRAASQDLA

       250        260        270        280        290        300
AIWAGFYIAG DPALAYGYAQ DQEPDAAGRI RNGALLRVYV PASSLPGFYR TSLTLAAPEA

       310        320        330        340        350        360
AGEVERLIGH PLPLALDAIT GPEEEGGRLE TILGWPLAER TVVIPSAIPT DPRNVGGDLD

       370        380
PSSIPDKEQA ISALPDYASQ PGKPPHHHHHHKDEL
```

Figure S1. Amino acid sequence of the targeted toxin DARPin-LoPE

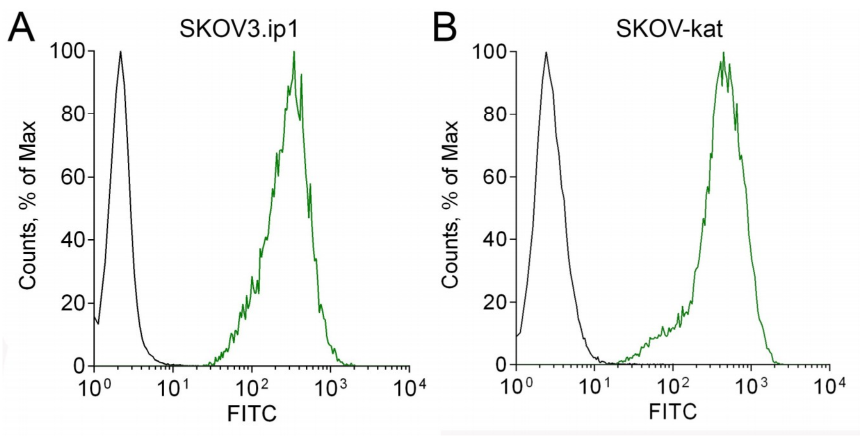

Figure S2. Analysis of surface content of the HER2 protein on cells. SKOV3.ip1 (**A**) and SKOV-kat (**B**) cells were stained with FITC-labeled anti-HER2 antibody (green line) or with FITC-labeled isotypic control (black line) and analyzed by flow cytometry.

A

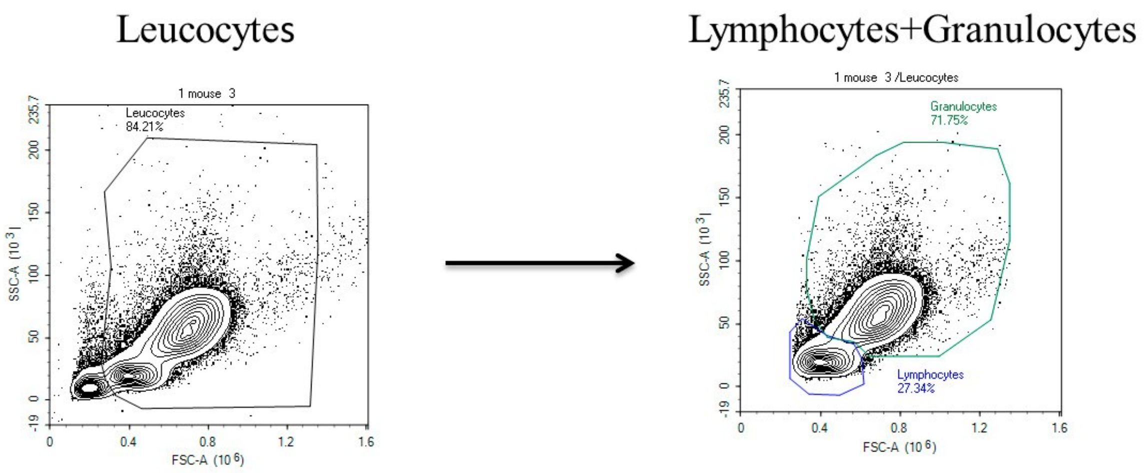

B

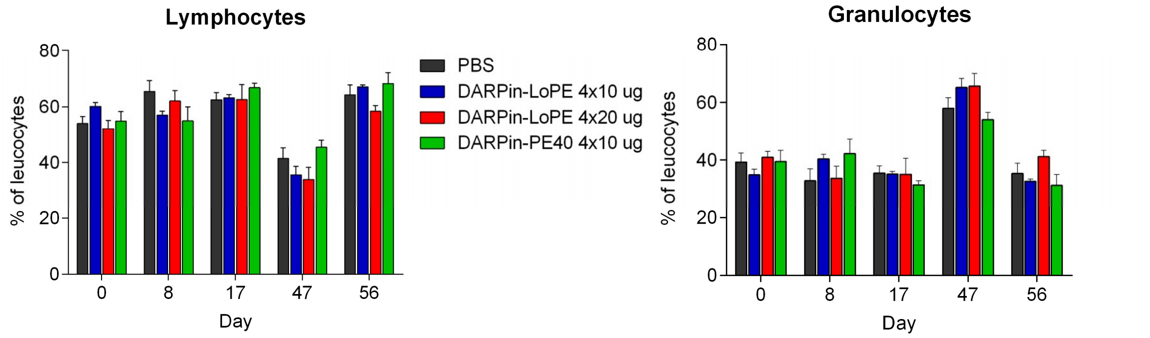

Figure S3. Analysis of leukocytes in the blood of animals by flow cytometry. (**A**) Gating strategy. The cells were separated from debris by forward and side scattering (SSC-A/FSC-A), then lymphocyte populations (small, non-granular) and neutrophil granulocytes (medium, granular) were isolated inside the leukocyte gates. For each population, the number of events and the proportion of this population among all leukocytes were recorded; (**B**) The proportion of lymphocytes (on the left) and granulocytes (on the right) in the general population of leukocytes before the start of injections (on day 0), then on days 8 and 17 after each course of injections started (i.e. on days 8, 17, 47 and 56 of the experiment). «PBS», mice treated with phosphate-buffered saline (control group); «DARPin-LoPE 4×10 μg», mice treated with 10 μg DARPin-LoPE for 4 doses every other day (40 μg per course, 80 μg total); «DARPin-LoPE 4×20 μg», mice treated with 20 μg DARPin-LoPE for 4 doses every other day (80 μg per course, 160 μg total); «DARPin-PE40 4×10 μg», mice treated with 10 μg DARPin-PE40 for 4 doses every other day (40 μg per course, 80 μg total). The data are represented as mean ±SEM.

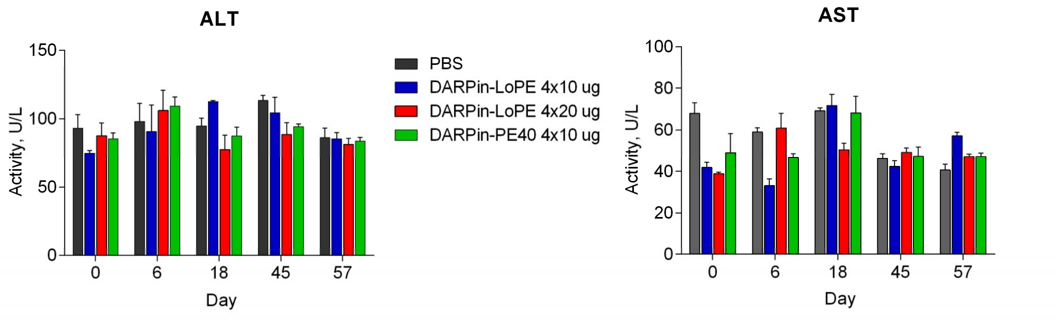

Figure S4. Analysis of the activity of alanine aminotransferase (ALT) and aspartate transaminase (AST) in the serum of mice by Reitman-Frankel method. ALT (on the left) and AST (on the right) activity before the start of injections (on day 0), then on days 6 and 18 after each course of injections started (i.e. on days 6, 18, 45 and 57 of the experiment). «PBS», mice treated with phosphate-buffered saline (control group); «DARPin-LoPE 4×10 µg», mice treated with 10 µg DARPin-LoPE for 4 doses every other day (40 µg per course, 80 µg total); «DARPin-LoPE 4×20 µg», mice treated with 20 µg DARPin-LoPE for 4 doses every other day (80 µg per course, 160 µg total); «DARPin-PE40 4×10 µg», mice treated with 10 µg DARPin-PE40 for 4 doses every other day (40 µg per course, 80 µg total). The data are represented as mean ±SEM.

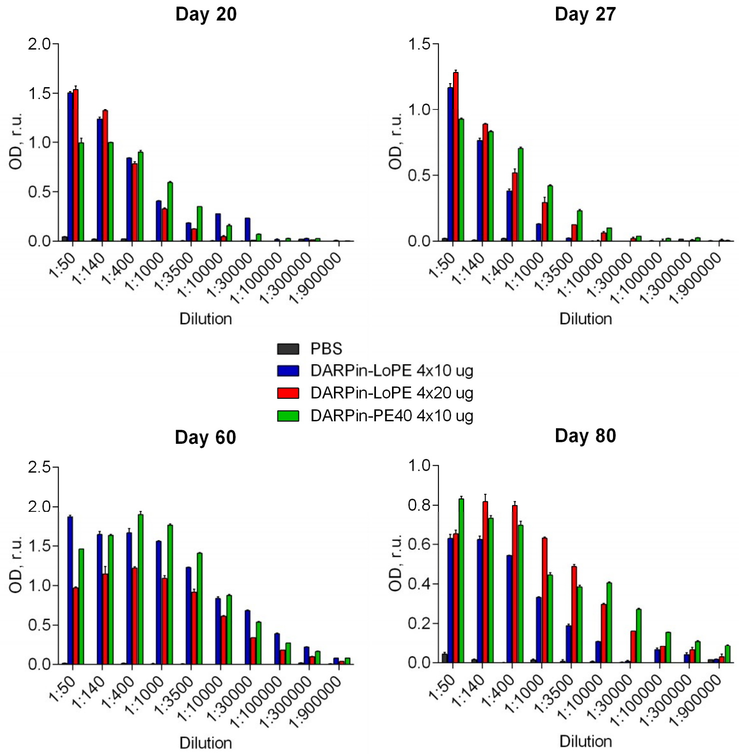

Figure S5. Analysis of the titer of antibodies specific to the targeted toxins by ELISA. Titers of antibodies two and three weeks after the first course ended (i.e. on days 20 and 27 of the experiment), and two and five weeks after the second course ended (i.e. on days 60 and 80 of the experiment) are presented. «PBS», mice treated with phosphate-buffered saline (control group); «DARPin-LoPE 4×10 µg», mice treated with 10 µg DARPin-LoPE for 4 doses every other day (40 µg per course, 80 µg total); «DARPin-LoPE 4×20 µg», mice treated with 20 µg DARPin-LoPE for 4 doses every other day (80 µg per course, 160 µg total); «DARPin-PE40 4×10 µg», mice treated with 10 µg DARPin-PE40 for 4 doses every other day (40 µg per course, 80 µg total). The data are represented as mean ±SEM.

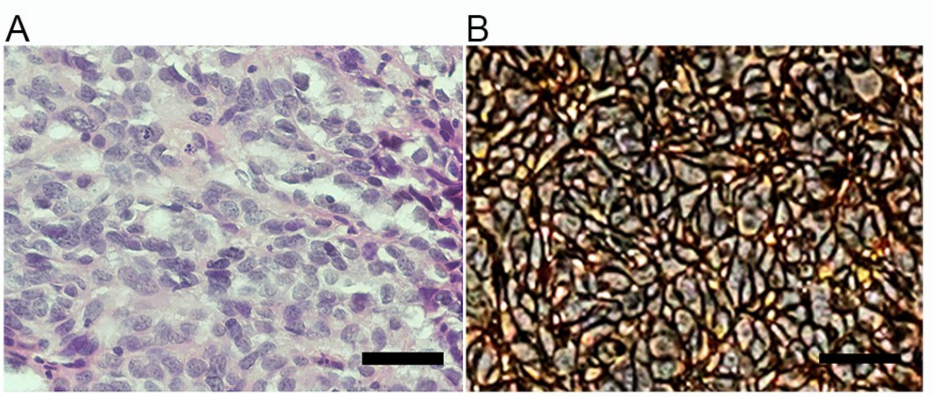

Figure S6. Characterization of tumor xenograft SKOVip-kat. (**A**) Staining of a tumor tissue section with hematoxilin and eosin; (**B**) Immunohistochemical staining of a tumor tissue section with HercepTest (Dako); strong brown coloring corresponds to HER2 overexpression. Scale bar – 50 μm.
